# Unveiling the hidden causal links: skin flora and cutaneous melanoma

**DOI:** 10.3389/fonc.2024.1451175

**Published:** 2024-12-11

**Authors:** Zexin Zhang, Wenfeng Wu, Jiajia Lin, Hongyi Li

**Affiliations:** ^1^ The Second Clinical School of Guangzhou University of Chinese Medicine, Guangzhou, China; ^2^ Department of Dermatology, The Second Affiliated Hospital of Guangzhou University of Chinese Medicine, Guangzhou, China

**Keywords:** causal relationship, skin flora, cutaneous melanoma, mendelian analysis, meta-analysis

## Abstract

**Objective:**

The presence of skin flora (SF) has been identified as a significant factor in the onset and progression of cutaneous melanoma (CM). However, the vast diversity and abundance of SF present challenges to fully understanding the causal relationship between SF and CM.

**Methods:**

A Two Sample Mendelian Randomization (TSMR) analysis was conducted to investigating the causal relationship between SF and CM. The Inverse-Variance Weighted (IVW) method was utilized as the primary approach to assess the causal relationship under investigation. Furthermore, an independent external cohort was employed to validate the initial findings, followed by a meta-analysis of the consolidated results. To address potential confounding factors related to the influence of SF on CM, a Multivariate Mendelian Randomization (MVMR) analysis was also conducted. Finally, a Reverse Mendelian Randomization (RMR) was conducted to further validate the causal association.

**Results:**

TSMR results showed that 9 SF have a causal relationship with CM in the training cohort. Although these 9 SF weren’t confirmed in the testing cohort, 4 SF remained significant in the meta-analysis after integrating results from both cohorts. MVMR analysis indicated that 3 SF were still significantly associated with CM after accounting for the interactions between different SF in the training cohort. No reverse causal relationship was identified in RMR analysis.

**Conclusion:**

A total of 9 SF were identified as having a potential causal relationship with CM; however, a large randomized controlled trial is needed to verify these results.

## Introduction

Cutaneous melanoma (CM) is a highly malignant tumor that originates from melanocytes in the basal layer of the epidermis, often presenting as irregular, dark-pigmented lesions on the skin surface (1). Globally, the incidence and mortality rates of CM are rising each year. In 2020, approximately 325,000 new cases of melanoma and 57,000 deaths were reported worldwide (2, 3). The prognosis of CM can vary significantly depending on factors such as age of onset, tumor location, and pathological type (4). Research shows that lymphatic metastasis can occur in the early stages of CM, drastically reducing survival rates. As the number of affected lymph nodes increases, the 5-year survival rate for patients plummets to around 10% (5). CM is highly aggressive and invasive, accounting for up to 90% of skin cancer-related deaths, making it the most lethal skin malignancy (6). Given its profound public health and economic impact, it is essential to identify prognostic risk factors for CM and explore novel treatment strategies.

At present, the specific pathogenesis of CM has not been elucidated. Related studies have shown that ultraviolet radiation exposure (Ultraviolet Radiation, UVR, including natural sunlight and artificial lighting systems) is the main risk factor for the occurrence of CM. Additional factors such as age, genetics, geographical location, and a history of skin cancer also contribute to CM incidence (7). The skin, as the largest organ of the human body, hosts a vast ecological niche for diverse microorganisms (8). Skin flora (SF) refers to the microbiome of bacteria, fungi, and viruses that colonize the skin, influencing human health to varying degrees (9). Disruption in the balance and homeostasis of SF have been kinded to several skin diseases, such as psoriasis (10) and acne (11). Consequently, alterations in SF composition hold potential for new diagnostic and therapeutic approaches for skin diseases (12).

Although SF has been showed to be closely related to certain diseases, its relationship with CM remains underexplored. UVR exposure, including ultraviolet A (UVA) and ultraviolet B (UVB), alters the composition of the skin microbiota. Among them, the bacterial group dominated by *Cyanobacteria* and *Fusobacteria* increased significantly, while *Lactic acid bacteria* and other bacterial groups decreased. This may be related to the physical effects that UVR brought about (13). A small sample study by Wang et al. found that *Propionibacterium acnes* can inhibit the survival of UVB-irradiated melanocytes by increasing cell apoptosis. At the same time, the skin commensal bacteria *Staphylococcus epidermidis* and its by-product Lipoteichoic Acid (LTA) induce the upregulation of TRAF1, CASP14, CASP5 and TP73 promoting melanocyte survival (14). In contrast, Nakatsuji et al. found that intravenous infusion of 6-N-hydroxyaminopurine (6-HAP) produced by *Staphylococcus epidermidis* in mice can inhibit the growth of B16F10 melanoma (15). The results of another small sample study suggested that there are differences in the skin microbiota of CM. Among them, acnes (*formerly Propionibacterium*), *Staphylococcus* and *Corynebacterium* are the most common bacterial genera, and the study showed that the diversity of microorganisms of melanoma samples decreased slightly (16). Michael et al. demonstrated the value of skin flora in the treatment of CM by constructing *Staphylococcus epidermidis* expressing tumor antigens and inducing T cell responses to limit melanoma growth (17). These findings suggest a possible association between CM and skin microbiota, yet no epidemiological or clinical studies have definitively established a causal link. Thus, exploring the causal relationship between skin microbiota and CM is crucial.

However, observational studies are limited in their ability to control for confounding factors and cannot fully avoid the effects of reverse causality, making it challenging to determine true associations between exposures and outcomes (18). While randomized controlled trials (RCTs) are the gold standard for establishing causal relationships, they often face obstacles such as high costs and implementation difficulties (19). Mendelian randomization (MR) is an epidemiological research method based on Mendel’s law of independent distribution, which uses genetic variation (usually single nucleotide polymorphisms, SNPs) as a tool for natural random assignment. Simulated randomized controlled trials have been widely used in recent years to reveal causal relationships between environmental exposures and disease outcomes (20, 21). In this study, a Two-Sample Mendelian Randomization (TSMR) analysis was performed using a large-scale genome-wide association study (GWAS) dataset to investigate the role of skin flora in the development of CM. The goal was to explore potential causal relationships between skin flora and CM, providing new insights into CM prevention and treatment.

## Materials and methods

### Data acquire and process

**Figure 1:**
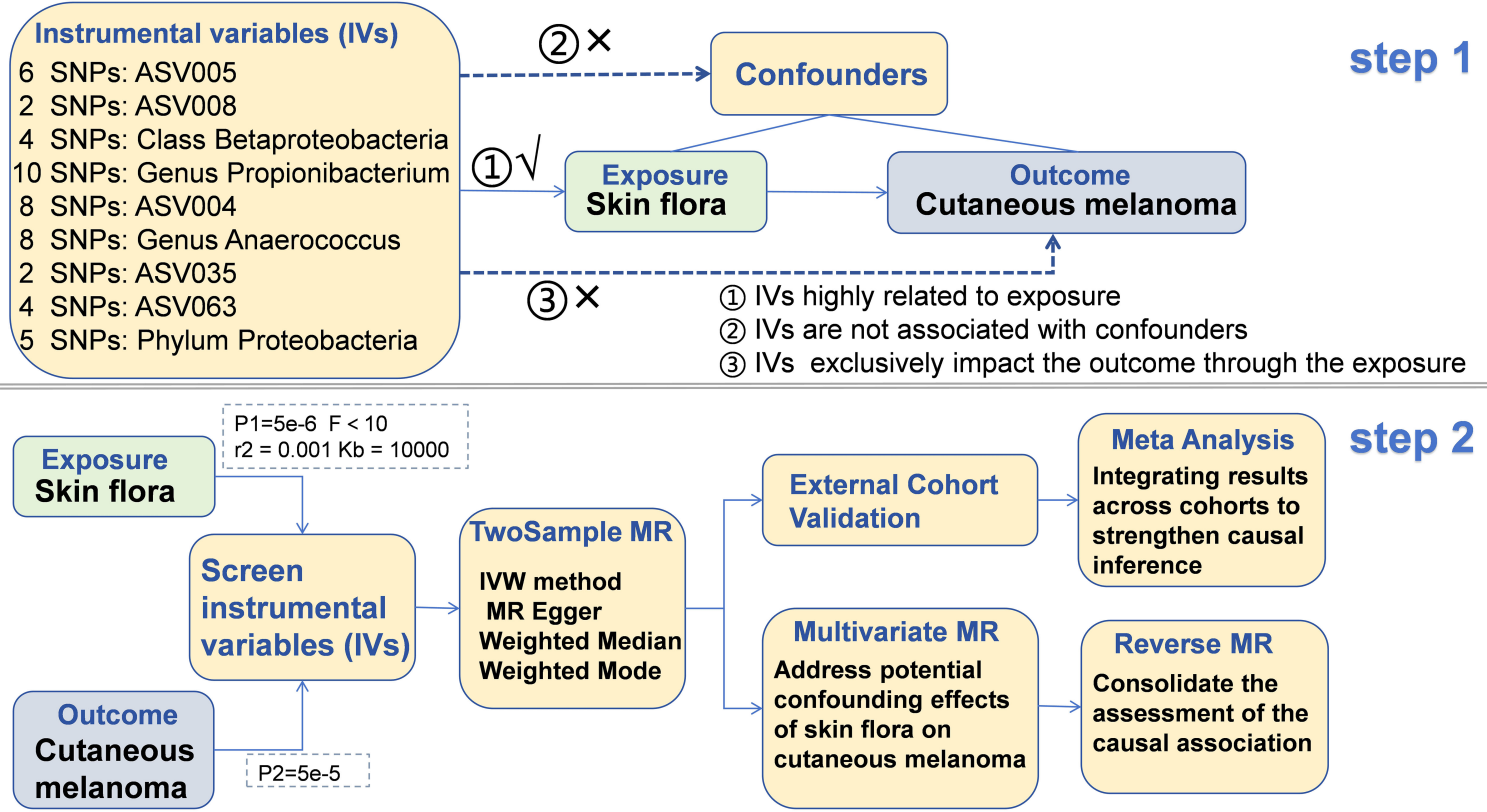
The flow of this study.

The SNP data of skin flora (SF) and cutaneous melanoma (CM) were obtained from European population. The GWAS summary data of SF was derived from the data published by Moitinho-Silva L et al. in 2022 (9). In this study, the authors conducted a large-scale Genome-Wide Association Study (GWAS) on two population-based German cohorts. These 296 SF originate from different parts of the body and are divided by skin properties into dry, moist and sebaceous skin. The flow of this study was showed in **Figure 1**.

### Screening of instrumental variables

Initially, by setting the threshold of P1 5e-6 was for single nucleotide polymorphisms (SNPs), instrumental variables (IVs) strongly correlated with SF were screened. SNPs demonstrating higher levels of significance were considered to be closely linked to the heritability of SF. Additionally, an F test was performed on each IV to eliminate those with weak correlations. The F test equation, given by F = (Beta/Se)^2, includes the impact size (Beta) of independent variables on SF and the standard error (Se) linked to Beta. The study did not include IVs with F test values lower than 10. Furthermore, an assessment of linkage disequilibrium was performed on the IVs. Linkage disequilibrium in genetics refers to the probability of alleles from multiple gene loci co-occurring on a single chromosome at a frequency higher than expected by chance. These occurrences are not conducive to MR analysis. To address this potential bias, a threshold of r2 = 0.001 and Kb = 10000 was set.

The IVs of CM are extracted simultaneously using the IVs of the SF after undergoing a screening process. A significance threshold of P2 = 5e-5 is established for the IVs of the outcome to remove highly correlated variables. Proxy tools are not introduced for IVs that are absent in the CM dataset to uphold the precision and dependability of the findings. Following this, the data of IVs from the SF and CM are merged, and palindromic SNPs are excluded.

### Two sample MR analysis

The TwosampleMR package in the R language was employed to perform a comprehensive MR analysis on the combined dataset. The MR analysis incorporated four different methods, namely the Inverse Variance Weighted (IVW), MR Egger, Weighted Median, and Weighted Mode. The findings derived from the IVW method were predominantly utilized for assessment. It is worth noting that the IVW method is advantageous for detecting bias even when invalid instrumental variables (IVs) are present (22). On the other hand, the MR Egger method incorporates an intercept to evaluate and address horizontal pleiotropy in IVs (23). For Weighted median, this estimate is consistent even if up to 50% of the information comes from invalid tool variables. In the simulation analysis, it is proved that it has better class 1 error rate than IVW method (24). The weighted mode model employs the reciprocal of the result variance as weights, thereby assigning greater significance to SNPs with smaller variances in the estimation process (25). In order to ensure the reliability of the findings, only results exhibiting consistent Beta direction across all four analysis methods were included.

### Multivariate Mendelian Randomization analysis

In order to improve comprehension of the role of different SF in CM and strengthen the credibility of TSMR results, Multivariate Mendelian Randomization (MVMR) analysis was utilized to investigate the potential association between SF and CM (26). Initially, shared SNPs present in multiple SF types were identified and subsequently extracted from the dataset. After removing linkage disequilibrium, the isolated SNP data underwent MVMR analysis. The IVW method, similar to TSMR, serves as a tool for assessing the primary outcome of MVMR. In addition, **Supplementary Methods** such as MR Egger, Lasso, and Weighted median are utilized to bolster the reliability of the IVW method. Heterogeneity assessment is performed using the IVW method, while the detection of pleiotropic effects is achieved through the analysis of the Egger intercept and MR Presso.

### Verification based on testing cohort and meta-analysis

In order to reduce the bias impact of individual study results on the findings of MR analyses, a meta-analysis was performed using data from two separate cohorts of CM patients to ascertain the magnitude of the association between SF and CM. The meta-analysis predominantly utilized the IVW method for the analysis of the relationship between SF and CM. The presence of heterogeneity in the meta-analysis was evaluated through the I2 statistic, with a fixed effects model applied for I2 values of 50% or less and a random effects model utilized for I2 values exceeding 50%.

### Statistic analysis

Horizontal pleiotropy significantly impacts the validity of MR analysis findings by introducing the possibility of IVs exerting effects on outcomes through multiple genetic pathways, thereby contravening fundamental MR analysis principles. To identify and address horizontal pleiotropy, we employed Presso (27) and MR Egger methodologies to assess IVs for potential pleiotropic effects. IVs that passed both tests simultaneously were regarded as not having horizontal pleiotropy, and those with pleiotropic effects were excluded from the study. Heterogeneity testing was also carried out on IVs using the IVW and MR Egger methods, with IVs that showed heterogeneity being excluded from the analysis. Furthermore, heterogeneity testing was conducted on IVs using the IVW and MR Egger methods. After identifying heterogeneity, those IVs were excluded from the analysis.

Each independent variable underwent a leave-one-out sensitivity analysis to determine the potential effect of individual SNPs on the outcome. The Steiger test was employed to detect and remove SNPs demonstrating reverse causality, which could introduce bias into the interpretation of the association between exposure and outcome variables. Furthermore, a Reverse Mendelian randomization (RMR) analysis was conducted to confirm the results by examining the relationship between CM and SF.

## Results

### Characteristics of SNPs

A total of 2 CM queues were obtained from the GWAS summary data, and their IDs were finngen_R10_C3_MELANOMA_SKIN_EXALLC and ukb-saige-172.11 respectively. Among them, finngen_R10_C3_MELANOMA_SKIN_EXALLC was used as the training cohort, and ukb-saige-172.11 was used as the testing cohort.

ukb-saige-172.11 contains more than 400 million detected variants and 397, 762 sample sizes, including 2, 691 cases and 395, 071 controls. The populations are from European, and the gender includes males and females.

### Screening of instrumental variables

Based on the selection criteria mentioned earlier, SNPs were extracted from the exposure and outcome variables for this analysis. In the end, 9 SF were identified as having a causal relationship with CM. 6 SNPs came from ASV005, 2 SNPs came from ASV008, 4 SNPs came from Class Betaproteobacteria, 10 SNPs came from Genus Propionibacterium, 8 SNPs came from ASV004, 8 SNPs came from Genus Anaerococcus, 2 SNPs came from ASV035, 4 SNPs came from ASV063, 5 SNPs came from Phylum Proteobacteria were finally included in the MR analysis. The instrumental variables had F values greater than 10, which indicates they were not weak. The detailed information of IVs was showed in **Supplementary Table S1**.

### TSMR analysis

**Figure 2:**
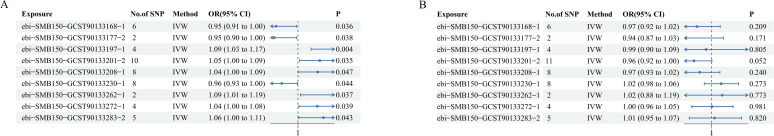
The IVW results of 9 skin flora against CM in training cohort and testing cohort. **(A)** finngen_R10_C3_MELANOMA_SKIN_EXALLC; **(B)** ukbsaige-172.11.

The IVW results indicated that a total of 9 SF are causally associated with CM, which included 3 protective factors and 6 risk factors. Specially, we found that ASV005 (OR: 0.952, 95% CI: 0.91 to 0.997, P value: 0.036), ASV008 (OR:0.948, 95% CI: 0.902 to 0.997, P value: 0.038) and Genus Anaerococcus (OR: 0.965, 95% CI: 0.931 to 0.999, P value:0.044) were the protective factors on CM, while Class Betaproteobacteria (OR: 1.095, 95% CI: 1.029 to 1.166, P value: 0.004), Genus Propionibacterium (OR: 1.048, 95% CI: 1.003 to 1.094, P value: 0.035), ASV004 (OR: 1.043, 95% CI: 1.001 to 1.088, P value: 0.047), ASV035 (OR: 1.093, 95% CI: 1.006 to 1.187, P value: 0.037), ASV063 (OR: 1.042, 95% CI: 1.002 to 1.084, P value: 0.039) and Phylum Proteobacteria (OR: 1.056, 95% CI: 1.002 to 1.112, P value: 0.043) were the risk factors for CM. Exposure factors are represented by their corresponding ID, the blue line segment represents the 95% confidence interval (CI) of the exposure factor, and the dot on the line segment represents the corresponding OR value. (**Figure 2A**, **Supplementary Table S2**). This outcome was also evident in the scatter plot generated from MR analysis. The scatter plot illustrates the influence of each SNP on 9 SF and CM, allowing for a visual representation of the impact of exposure on the outcome. The different colored lines represent different approaches, and they sum up all of the SNPs’ contributions to CM, depicting an upward OR downward trend according to the size of the OR value. If the line extends from the bottom left to the top right, SF is a risk factor for CM; if the line extends from the top left to the bottom right, SF is a protective factor for CM (**Supplementary Figure S1**). The Forest plot presented the MR effect size of the 9 SF and CM for each IVs. MR Egger and IVW methods were used to calculate the MR effect sizes of all IVs, which were then displayed in red line segment (**Supplementary Figure S2**). Heterogeneity testing indicated that the included IVs were homogeneous, and the distribution on the funnel plot was symmetrical based on the method of IVW and MR Egger (**Supplementary Figure S3**, **Supplementary Table S3**). A leave-one-out sensitivity analysis, which systematically eliminated each independent variable, demonstrated the robustness of the results (**Supplementary Figure S4**). Additionally, no evidence of a reverse causal relationship was observed between 9 SF and CM.

### Verification based on testing cohort

In order to verify the reliability of the training cohort results, TSMR analysis was performed using the same parameters in the testing cohorts. Similar to **Figure 1A**, the exposure factor is displayed by its corresponding ID, the blue line segment represents the 95% CI of the exposure factor, and the dot on the line segment represents the corresponding OR value. However, none of the SF in the testing cohort has been verified to be causally associated with CM (**Figure 2B**).

### Meta-analysis based on inverse-variance weighted method of training cohort and testing cohort

**Figure 3:**
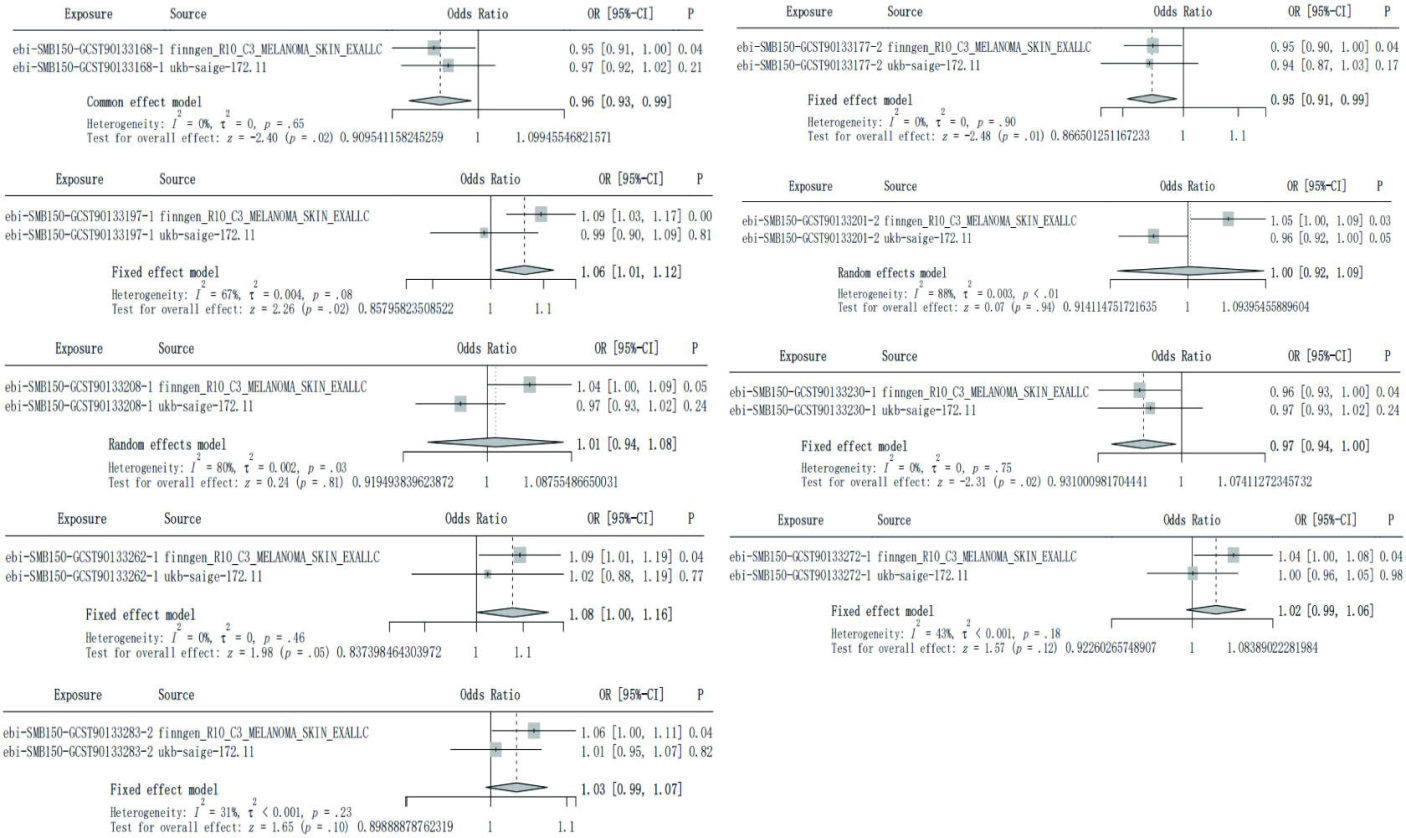
Meta-analysis integrated the IVW results of 9 skin flora against CM in training cohort and testing cohort.

Although none of the SF in testing cohorts has been verified to be causally associated with CM, after integrated the results of IVW method to conduct meta-analysis, a total of 5 SF showed potential causal relationship on CM with significant differences. Source 1 refers to finngen_R10_C3_MELANOMA_SKIN_EXALLC, Source 2 refers to ukb-saige-172.11. Similar to **Figure 1**, the exposure factor is displayed by its corresponding ID, the black line segment represents 95% CI of the exposure factor, and the diamond represents 95% CI obtained by integrating the training group and the testing group (**Figure 3**).

### MVMR analysis

**Figure 4:**
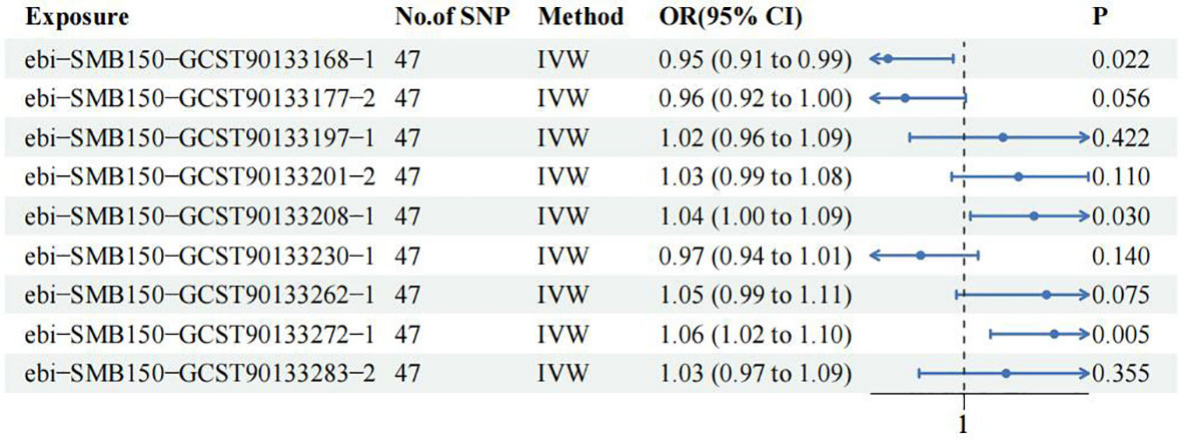
Multivariate Mendelian Randomization analysis among 9 skin flora against CM in training cohort.

In order to further clarify the role of 9 SF in CM and consolidate the reliability of TSMR and meta-analysis results, MVMR was used to analyze SF with positive results. 47 shared IVs were extracted among the 9 SF. The results of TSMR from 3 SF remained stable in the MVMR analysis in training group. Among them, *ASV005* (IVW OR: 0.951, 95% CI: 0.911-0.993, P value:0.022) was a protective factor for CM, while *ASV004* (IVW OR: 1.045, 95% CI: 1.004-1.087, P value:0.03) and *ASV063* (IVW OR: 1.058, 95% CI: 1.017-1.101, P value:0.005) were the risk factors for CM (**Figure 4**). MR Egger and IVW analyses showed no heterogeneity in the instrumental variables. The Egger intercept was close to zero, and the P value was above 0.05, which indicates a lack of horizontal pleiotropy (**Supplementary Table S4**). This results were confirmed in MR Presso test (**Supplementary Table S5**).

## Discussion

The skin acts as a physical barrier between the human body and the external environment. It offers a niche for the commensal microbiota while simultaneously shielding against the incursion of alien pathogens (28). Disruptions in the skin flora (SF) could potentially influence the development of skin tumors through direct cellular damage (29), modulation of host defense mechanisms (29), and alterations in the tumor immune microenvironment (30). Additionally, toxins secreted by the SF have the capacity to induce oncogenic mutations via DNA damage (31). The research by Hoste et al. (32)demonstrated that SF induces the production of the inflammatory factor HMGB1 through the pathway of Toll-like receptor 5 (TLR-5) recognition of bacterial flagellin. This process may facilitate the transition from skin injury or chronic inflammation to cutaneous malignancy. SF impacts the formation and stability of the skin barrier (33), and its imbalance may exacerbate UV radiation-induced DNA damage and reactive oxygen species (ROS) production (34). This leads to an enhanced inflammatory response and increased cell proliferation, ultimately disrupting the skin barrier and promoting skin tumorigenesis (34). In conclusion, the role of the skin microbiota should not be overlooked in investigations into the pathogenesis of skin tumors. CM, as a skin malignancy with a high risk, exhibits associations between their onset and progression with dysregulation of the SF ecosystem (35, 36). However, the precise causal relationships remain to be elucidated.

A total of 9 SF showed causal relationship with CM in TSMR results in this study. The results showed that ASV005, ASV008 and *Genus Anaerococcus* were the protective factors on CM, while *Class Betaproteobacteria*, *Genus Propionibacterium*, ASV004, ASV035, ASV063 and *Phylum Proteobacteria* were the risk factors on CM. *Staphylococcus* has a bidirectional regulation of the occurrence risk of CM.

*Proteobacteria* and *β-Proteobacteria* are considered risk factors for CM (35). They all belong to the group of Gram-negative bacteria, characterized by an outer wall containing a specific component known as lipopolysaccharide (LPS). Currently, there is limited research on the role of *Proteobacteria* and *β-Proteobacteria* in skin diseases. However, some studies have suggested that their proliferation may promote inflammation or facilitate the invasion of external pathogens (37, 38). Furthermore, an increase in *Proteobacteria* abundance has been proposed as a potential diagnostic marker for flora imbalance and disease susceptibility (39). LPS derived from these bacteria can activate intestinal mucosal immunity, leading to local and systemic inflammation as well as metabolic dysfunction (40). Toll-like receptor 4 (TLR4) expression has been observed in 90% of human primary melanoma lesions and 93% of metastatic lesions (41). As a ligand for TLR4, LPS promotes the proliferation and migration of TLR4-positive melanoma cells through signal transduction mediated by transcriptional activator 3 (STAT3) activation (42). Therefore, it is plausible that *Proteobacteria* and *β-Proteobacteria* indirectly activate STAT3 via LPS signaling pathway exacerbating CM progression.

*Corynebacterium* is considered a significant risk factor for CM (43). According to statistical data, *Corynebacterium* is the most frequently detected or second most frequently detected bacterial genus in stage III/IV melanoma patients. Additionally, coryne-positive patients exhibit higher levels of IL-17 positive cells compared to coryne-negative patients (43). IL-17 can stimulate the production of IL-6, which subsequently activates STAT3 and upregulates genes associated with cell survival and angiogenesis (44). Therefore, it is possible that *Corynebacterium* may contribute to the progression of CM by promoting the IL-6-STAT3 pathway.

According to the analysis, we considered *Fingolderia magnus* to be a risk factor for CM. Formerly known as *peptostreptococcus magnus*, *Fingolderia magnus* is considered one of the most pathogenic opportunistic pathogens capable of causing severe infections in various anatomical sites including bone, joint, lung, skin and soft tissue, as well as infective endocarditis (45, 46). The key virulence factors associated with *E. grandis* pathogenesis encompass protein L, Surface-associated protein F. magna adhesion factor (FAF), Subtilisin-like extracellular serine protease (SufA), Sortase dependent pili, Peptostreptococcal albumin-binding protein (PAB). Its pathogenic mechanisms involve inhibiting biofilm formation, evading host immune defense mechanisms and inducing secretion of pro-inflammatory factors (47, 48). It is plausible that *Fingoldelia magnus* may augment the risk of CM through similar immune evasion and proinflammatory pathways.

*Propionibacterium granulosa*, *Propionibacterium acnes*, and *Propionibacterium Feideri* all belong to the *genus Propionibacterium* and *Cutibacterium* (49). The findings of this study suggested that *propionibacteria* are a risk factor for the pathogenesis of CM, while g*ranular propionibacterial species* have a protective effect on CM, which may be attributed to their differential mechanisms of action. *Propionibacterium acnes* promotes the synthesis of pro-inflammatory cytokines such as IL-1, IL-6, TNF-α, IL-12 and IL-18, leading to an inflammatory response (50, 51). Previous studies have indicated the involvement of *proinflammatory* mechanisms by *propionibacteria* in prostate cancer pathogenesis and its high abundance in prostate cancer (52, 53). It is plausible that *propionibacteria* exacerbate CM pathogenesis through similar proinflammatory reactions. Genomic studies have demonstrated distinct separations between *propionicbaterrium granulosus* and other species within the *genus Propionicbaterrium* suggesting significant differences in their potential functions on skin health (54). Currently limited research exists regarding the mechanism of action for *Propionicbaterrium granulosus*. However, some studies have reported the discovery of an endogenous extracellular nuclease BmdE secreted by this bacterium which can degrade biofilms produced by *Propionicbaterrium acnes* both *in vivo* and *in vitro* (55). These results propose a potential novel competitive mechanism between *P. acnes* and *P. granulosus* possibly related to diverse effects exerted by *P. granulosus* compared with other members within the genus.

The findings of this study demonstrated that *staphylococcus* exerts a bidirectional regulatory effect on the risk of CM (56). *Staphylococcus epidermidis* is commonly considered as a commensal microorganism in the skin microbiota, contributing to the maintenance of skin barrier homeostasis, promotion of wound healing, enhancement of skin immunity, and inhibition of pathogen infection through the production of protective ceramides (57). 6-HAP, a molecule produced by *Staphylococcus epidermidis* that inhibits DNA polymerase activity, exhibits selective growth inhibition against tumor cell lines. Intravenous administration of 6-HAP in mice has been shown to suppress B16F10 melanoma growth, suggesting a potential protective role for Staphylococcus in CM (15). However, both *Staphylococcus epidermidis* and its by-product LTA can promote melanocyte survival by inducing upregulation of TRAF1, CASP14, CASP5 and TP73 genes. This suggested that *staphylococcus* may exacerbate disease progression in CM (14). Introduction of *Staphylococcus epidermidis* into germ-free mice has been demonstrated to restore normal IL-17A production - a chemokine potentially involved in tumor growth and anti-tumor immunity (58). In conclusion, the impact of *staphylococcus* on CM may be mediated through different metabolites; however, further investigation is required to elucidate the specific mechanisms involved.

The breast microbiome of healthy tissues and cancer-related tissues has been compared in studies, revealing a higher abundance of *anaerobes* in the former (59). A high level of bile acid metabolism generally indicates a better prognosis for breast cancer, with the high bile acid metabolism group exhibiting a greater abundance of anaerobes compared to the low bile acid metabolism group (60). These findings suggested potential disease protection mechanisms associated with *anaerobic* coccus. However, the current understanding of its protective mechanism in diseases remains unexplored. Therefore, the conclusions drawn from this study may offer new directions for future research on CM.

As we listed earlier, many studies have shown that there is a certain relationship between SF and CM. This study further provides a basis for consolidating this relationship by combining TSMR, MVMR and RMR. However, there are some limitations in this study, such as the heterogeneity of population background in different research cohorts. Some stratification factors, such as age and gender, were not disclosed in detail in the original study. Therefore, further randomized controlled trials are necessary to prove this conclusion.

## Conclusion

In conclusion, our study revealed that 9 SF have causal relationship with CM. Further large randomize control trail to verify this results is needed.

## Data Availability

The original contributions presented in the study are included in the article/**Supplementary Material**. Further inquiries can be directed to the corresponding author.

## Supplementary material

Supplementary Figure 1 The scatter plot of the effect distribution of all SNPs in training cohort.

Supplementary Figure 2 The forest plot of MR effect size of 9 skin flora against CM in training cohort.

Supplementary Figure 3 The funnel plot of distribution of all SNPs in training cohort.

Supplementary Figure 4 The Leave-one-out sensitivity analysis showed that no single SNP was found to have a significant impact on the outcome in training cohort.

Supplementary Table 1 The detail of the characteristics of SNPs in training cohort.

Supplementary Table 2 All MR results of 9 skin flora against CM in training cohort.

Supplementary Table 3 Heterogeneity test of 9 skin flora against CM in training cohort.

Supplementary Table 4 Pleiotropy test of 9 skin flora against CM on MR Egger in training cohort.

Supplementary Table 5 Pleiotropy test of 9 skin flora against CM on MR presso in training cohort.
