# Supplementary figures and images for: Unveiling the hidden causal links: skin flora and cutaneous melanoma
Source: Front Oncol. 2024 Dec 11;14:1451175. doi: 10.3389/fonc.2024.1451175 (PMC11668787; doi:10.3389/fonc.2024.1451175)

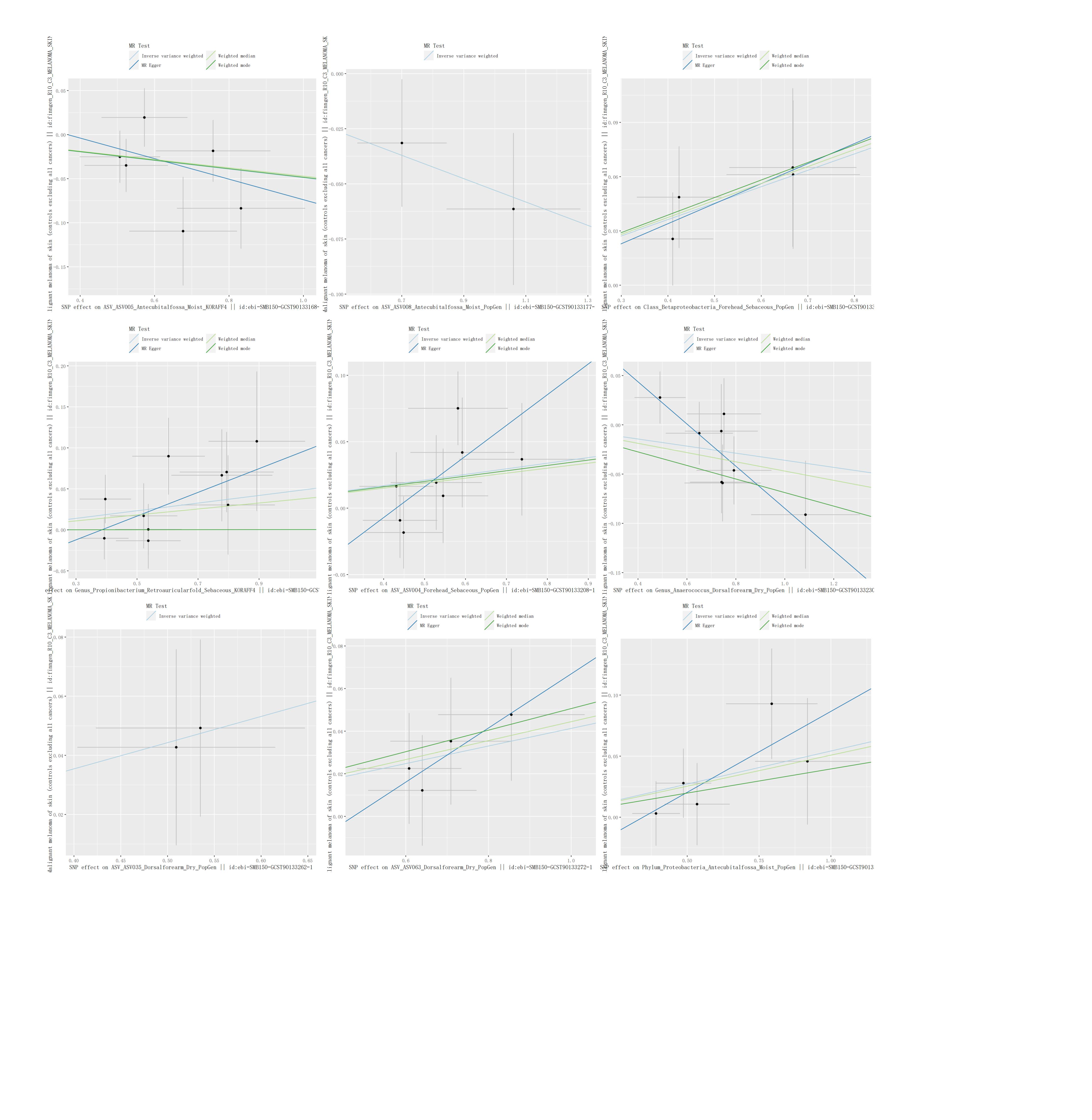

Supplement: Supplementary Figure 1 — The scatter plot of the effect distribution of all SNPs in training cohort. [file Image1.jpeg]

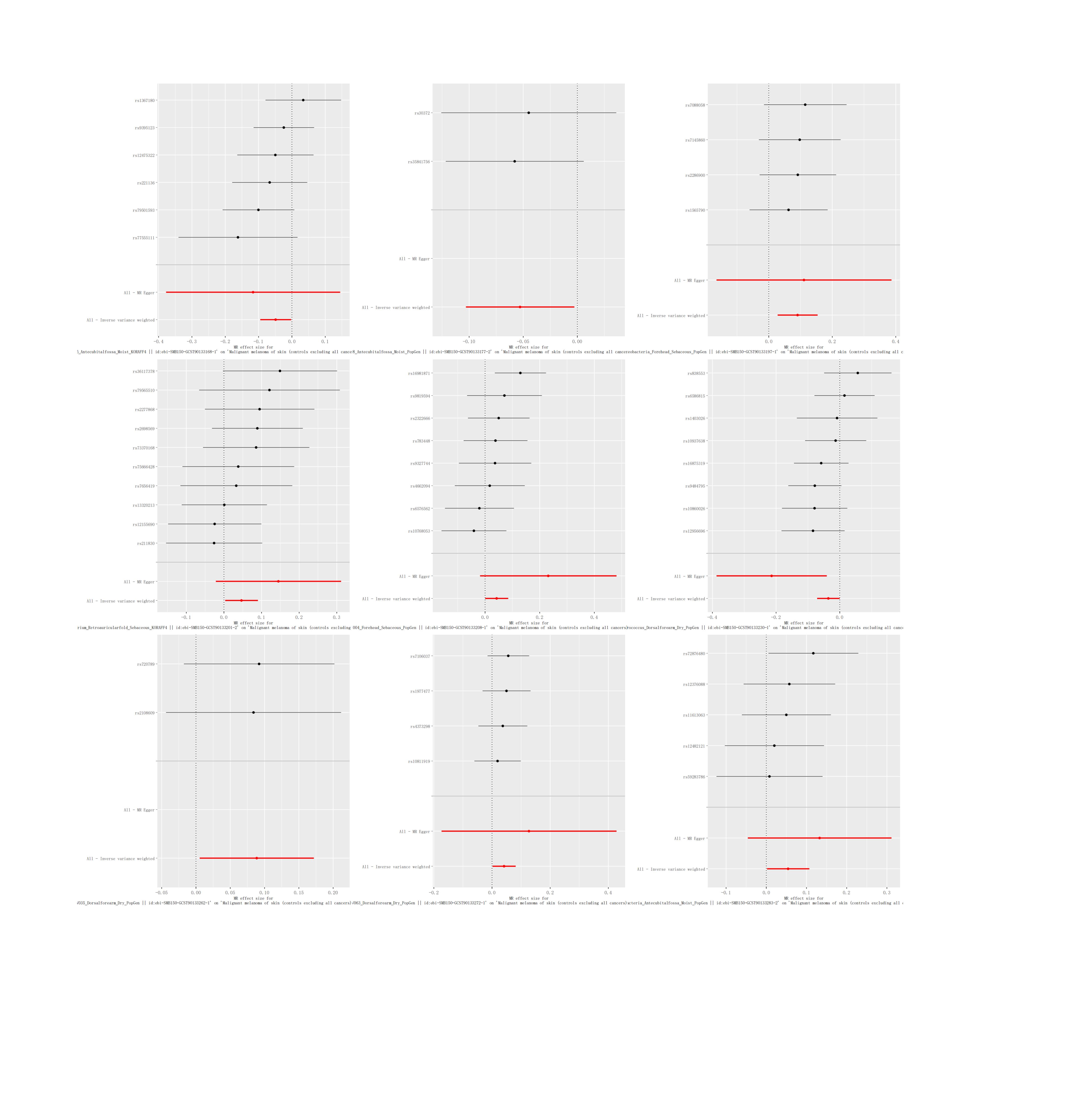

Supplement: Supplementary Figure 2 — The forest plot of MR effect size of 9 skin flora against CM in training cohort. [file Image2.jpeg]

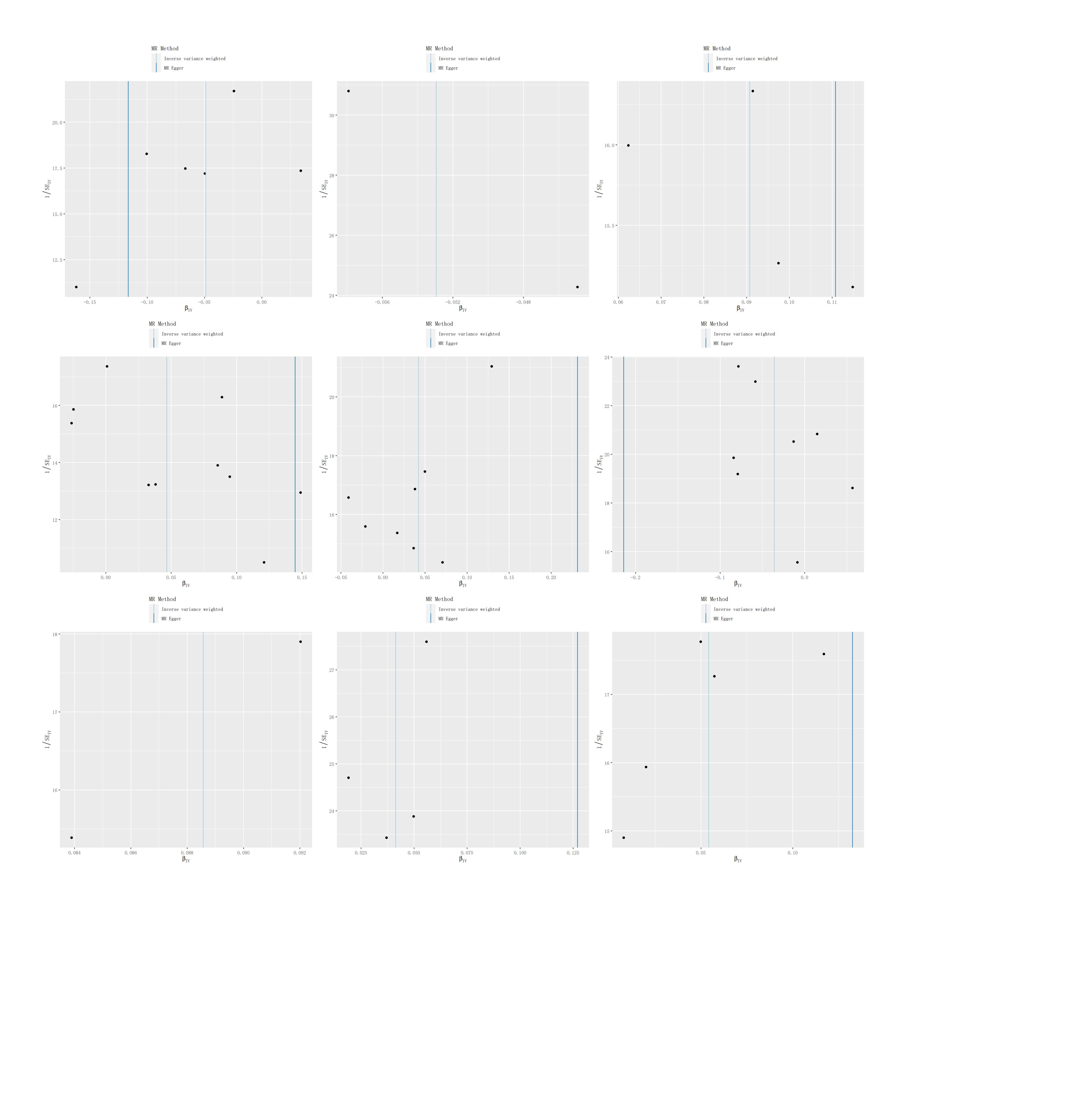

Supplement: Supplementary Figure 3 — The funnel plot of distribution of all SNPs in training cohort. [file Image3.jpeg]

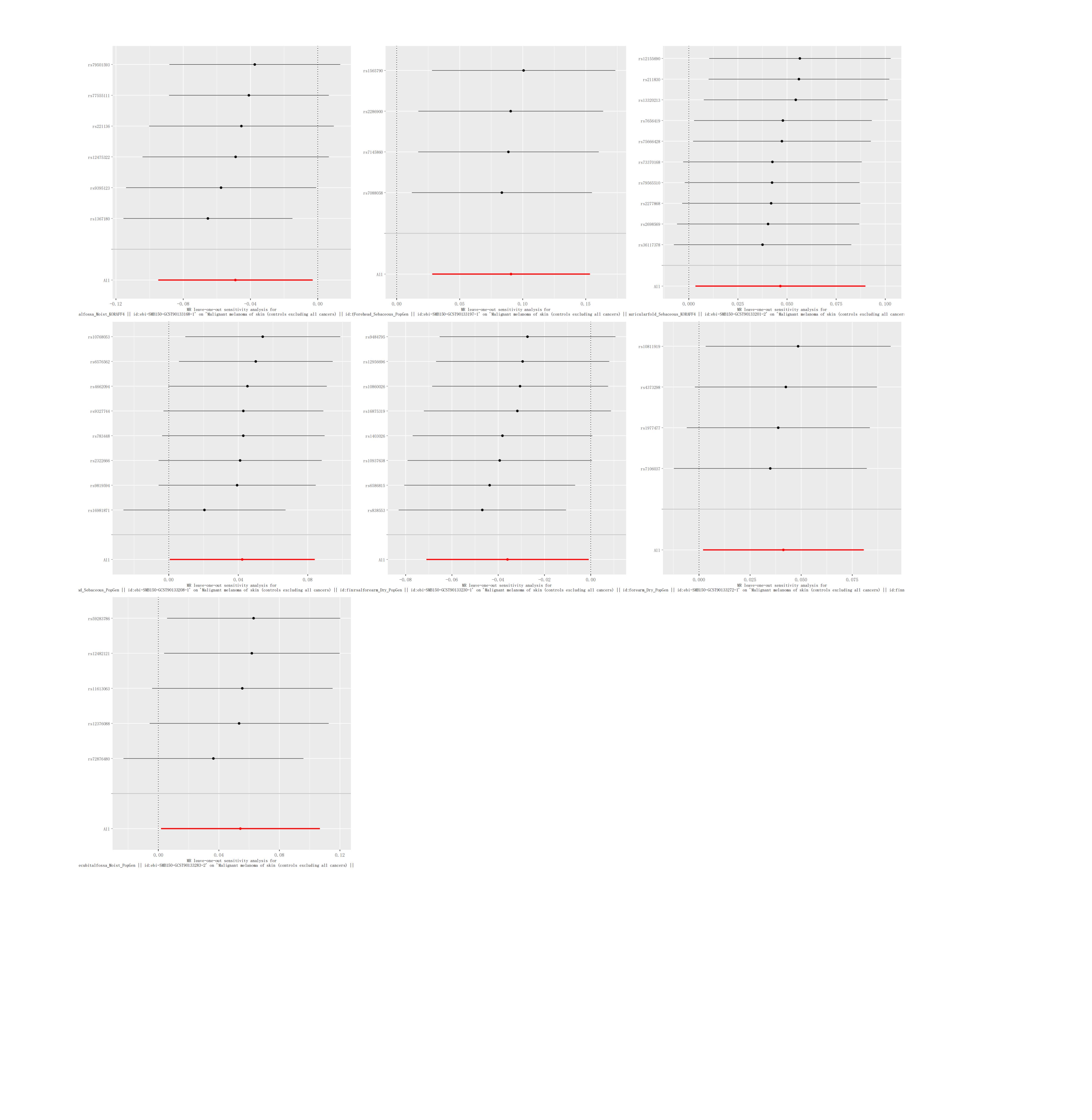

Supplement: Supplementary Figure 4 — The Leave-one-out sensitivity analysis showed that no single SNP was found to have a significant impact on the outcome in training cohort. [file Image4.jpeg]
